# Acitretin-associated erectile dysfunction: a case report

**DOI:** 10.1186/1757-1626-2-210

**Published:** 2009-11-19

**Authors:** Marco Rossi, Michele Pellegrino

**Affiliations:** 1 Department of Pharmacology "Giorgio Segre", University of Siena, Strada delle Scotte 6, 53100 Siena, Italy; 2 Tuscan Regional Center for Pharmacovigilance, Florence, Italy; 3 Department of Dermatological Sciences, Department of Clinical Medicine and Immunological Sciences, University of Siena, Policlinico S. Maria alle Scotte, 53100 Siena, Italy

## Abstract

**Introduction:**

Two cases of impotence following a treatment with etretinate have been reported in the literature. Acitretin is the principal active metabolite of etretinate. We report a case of erectile dysfunction associated with the use of acitretin.

**Case presentation:**

A 39-year-old Caucasian man referred his incapacity to reach and maintain a penile erection during a course of acitretin for the treatment of a severe form of psoriasis. Physical examination, laboratory findings and psychological analysis did not reveal any abnormalities. Two weeks after the withdrawal of acitretin, the patient reported a normalization of his sexual activity.

**Conclusion:**

Retinoids have been associated with male reproductive system dysfunctions in human and animal studies. Clinicians should be aware of the possibility of acitretin-induced erectile dysfunction.

## Introduction

Acitretin is a synthetic aromatic retinoid used to treat dermatologic diseases, especially psoriasis and related skin disorders. The major adverse effects observed with retinoids treatment have been essentially embryotoxicity, teratogenicity, mucocutaneous reactions, pruritus, hypertriglyceridemia, hypercholesterolemia, increases in liver transaminase concentrations and bone abnormalities. Acitretin is the principal active metabolite of etretinate, formerly approved for psoriasis, but withdrawn from the market because of its undesirable pharmacokinetic profile. We report a case of erectile dysfunction following a treatment with acitretin.

## Case presentation

A 39-year-old Caucasian man affected by psoriasis from approximately two years presented to our outpatient department with a worsening of his psoriatic lesions previously controlled by topical steroids and ultra-violet therapy.

The patient had no history of tobacco or alcohol use and he had not undergone major surgery. He was not taking drugs and his routine blood tests, hepatitis markers, coagulation parameters, thyroid function and chest radiography were normal.

After histological examinations, he started a treatment with acitretin 25 mg/die. His psoriatic plaques resolved after two months and the dose was reduced to 20 mg/die.

In a follow-up visit the patient reported his incapacity to reach and maintain a penile erection sufficient to perform a sexual act from approximately 45 days. The patient also referred the absence of morning erections. Physical examination of the external genital organs revealed neither fibromatous plaques, nor signs of vascular, endocrine or neurological diseases. Laboratory findings did not show any alterations and clinical symptoms of depression were not present.

We hypothesized a role of acitretin in the genesis of the erectile dysfunction, thus we decided to withdraw the drug with the consent of the patient. After two weeks he reported a normalization of his sexual activity and decided not to start a new course of acitretin despite the worsening of his psoriatic lesions.

## Discussion

A literature search using PubMed did not reveal any case reports on the association between acitretin use and erectile dysfunction. However, we retrieved two cases of impotence related to etretinate therapy. Etretinate has been detected in the plasma of patients receiving acitretin, because it is readily esterified in vivo to produce etretinate, especially in the presence of ethanol [1].

In the first case, the authors associated the erectile dysfunction with the use of etretinate in a 37-year-old patient with symptoms of depression on the basis of a positive dechallenge [2].

The second case, described in a 40-year-old patient without symptoms of depression, was confirmed by a positive rechallenge [3].

Acitretin has been associated to erectile dysfunction during spontaneous surveillance. We retrieved 2 cases in the database of the Netherlands Pharmacovigilance Centre LAREB [4] and 6 cases in the database of the Medical and Healthcare products Regulatory Agency of UK [5].

Other retinoids were involved in cases of erectile dysfunction. During a prospective study designed to evaluate the efficacy and safety of isotretinoin in the treatment of acne, six male patients referred difficulties in maintaining penile erection in association with clinical symptoms of depression [6]. One case of ejaculatory disorder related to the use of isotretinoin has been reported [7]. Retinoids activity on male reproductive system was observed in animal studies. Single neonatal treatment with retinol dramatically reduced the sexual activity of adult male rats [8]. In animals treated with retinoids, testicular atrophy with spermatogenetic arrest was described [9].

Our diagnosis is based only on the anamnesis and a positive dechallenge, as the patient preferred not to contact an urologist for his dysfunction and he did not agree to resume the treatment with acitretin, therefore a rechallenge was not possible.

On the basis of the literature review, we hypothesize a class effect of retinoids on erectile function. According to the animal studies [8,9], erectile dysfunction associated with retinoids therapy could be caused by a direct action on the male reproductive system, although a role of depression can not be excluded [10].

Patients are reluctant to report sexual dysfunctions during their contacts with practitioners, therefore it is likely that the incidence of similar effects may result underestimated.

## Conclusion

To our knowledge, no case of acitretin-associated impotence has been published to date. The case described has been reported to the Italian Pharmacovigilance System and to the manufacturer of the drug.

Prescribers of the drug, particularly dermatologists, should be aware of the possibility of such adverse reactions during a treatment with acitretin.

## Consent

Written informed consent was obtained from the patient for publication of this case report. A copy of the written consent is available for review by the Editor-in-Chief of this journal.

## Competing interests

The authors declare that they have no competing interests.

## Authors' contributions

MP analyzed and interpreted the patient's clinical data. MR performed the literature review and was the major contributor in writing the manuscript. All authors read and approved the final manuscript.
